# Emission Properties of Diblock Copolymers Composed of Poly(ethylene glycol) and Dense 1,2,3-Triazole Blocks

**DOI:** 10.3390/polym11071086

**Published:** 2019-06-26

**Authors:** Yanqiong Yang, Asami Mori, Akihito Hashidzume

**Affiliations:** Department of Macromolecular Science, Graduate School of Science, Osaka University, 1-1 Machikaneyama-cho, Toyonaka, Osaka 560-0043, Japan

**Keywords:** emission properties, diblock copolymer, dense 1,2,3-triazole block, poly(ethylene glycol) block, copper(I)-catalyzed azide–alkyne cycloaddition polymerization

## Abstract

This article describes a new block copolymer (EG*m*-*b*-AP*n*, where *m* and *n* denote the degrees of polymerization) of poly(ethylene glycol) (PEG) and poly(1,4-(1-*H*-1,2,3-triazolylene)methylene) prepared by copper(I)-catalyzed azide–alkyne cycloaddition (CuAAC) polymerization of 3-azido-1-propyne (AP) in the presence of PEG carrying a propargyl moiety. The EG*m*-*b*-AP*n* samples are well soluble in polar organic solvents. Unexpectedly, we observed that solutions of EG*m*-*b*-AP*n* in *N*,*N*-dimethylformamide emitted fluorescence. We systematically studied absorption and emission properties of the block copolymers. The experimental data have exhibited that AP*n* block is an intrinsic fluorophore. Interestingly, the emission of EG*m*-*b*-AP*n* can be easily tuned from ultraviolet to green fluorescence by changing the excitation wavelength. This enables fine-tuning of its optical property without the need of changing the chromophore. Moreover, the block copolymers show a fluorescence response to metal ions (e.g., Cu^2+^). Our discoveries contribute to the fundamental understanding of the optical properties of dense triazole-based polymer and raise intriguing prospects for fabricating novel emissive triazole-based materials.

## 1. Introduction

1,2,3-Triazole is an intriguing nitrogen-rich aromatic heterocycle possessing a large dipole moment [1,2,3]. 1,2,3-Triazoles are formed from azides and alkynes by Huisgen cycloaddition [4,5], one of 1,3-dipolar cycloaddition reactions. It is known that Huisgen cycloaddition proceeds regioselectively to form 1,4-disubstituted-1,2,3-triazoles in the presence of copper(I) compounds [6,7,8]. This reaction is called copper(I)-catalyzed azide–alkyne cycloaddition (CuAAC), which provides a high efficiency even in the presence of various functional groups without protection. Consequently, CuAAC has been one of the most important reactions in “click chemistry” [9,10]. On the basis of the structural features [2,11], 1,2,3-triazole acts as noncovalent interaction sites through coordination bonding, π–π stacking, and dipole–dipole interaction [11,12,13,14,15]. Some examples have been reported on functional polymers containing 1,2,3-triazole units. Polymer-carrying 1,2,3-triazole moieties can be utilized for gelation [16], sensing [17], and control of clouding point [18] through coordination bonding and solubility control [14], controlled release [19], and tough network polymer [20] through π–π stacking. Therefore, polymers composed of denser 1,2,3-triazole moieties are highly promising for new functional polymers that exhibit characteristic interactions or associations.

In our previous work, we conducted CuAAC polymerization of 3-azido-1-propyne (AP) and 3-azido-1-butyne (AB) to form oligomers possessing a number of 1,2,3-triazole moieties linked through a carbon atom [21]. To the best of our knowledge, these oligomers of AP and AB are ones possessing the densest 1,2,3-triazole moieties in the backbone. The AP and AB oligomers are thus promising as functional polymers based on the properties of 1,2,3-triazole. The dense 1,2,3-triazole oligomers were insoluble in common organic solvents presumably because of strong dipole–dipole interaction, although they were well soluble in strong acids (e.g., concentrated sulfuric acid, nitric acid, and hydrochloric acid). This observation indicates that protonation makes the oligomers soluble. The AP oligomer was thus quarternized with methyl iodide to obtain an oligomer soluble in polar organic solvents [22]. Since the quarternized oligomer possesses dense quaternized 1,2,3-triazole moieties, it can be expected to be a precursor of polymeric ionic liquid [23,24,25]. 

Recently, we have synthesized a diblock copolymer (EG*m*-*b*-AP*n*, *m* and *n* denote the degrees of polymerization (DP)) of poly(ethylene glycol) (PEG) and dense 1,2,3-triazole blocks to investigate the properties of AP oligomer in solution. The EG*m*-*b*-AP*n* samples obtained were well soluble in water and other polar solvents. Contrary to our expectation, these diblock copolymer samples emitted fluorescence in *N*,*N*-dimethylformamide (DMF). EG*m*-*b*-AP*n* is fluorescent by itself although AP*n* block, in which 1,2,3-triazole moieties are connected through a methylene, is not conjugated. Since fluorescent polymers have been widely applied to important fields (e.g., molecular sensors [26,27,28], nonlinear optical materials [28,29], and organic electroluminescence [28,30]), a new type of fluorescent polymers should be developed. In this study, we have investigated photophysical properties of EG*m*-*b*-AP*n* by absorption and steady-state fluorescence spectroscopies. We have also discussed the fluorescence behavior of EG*m*-*b*-AP*n* by comparing the experimental data with the results of density functional theory calculation. We have further examined the fluorescence behavior of EG*m*-*b*-AP*n* responsive to external stimuli (i.e., metal ions). 

## 2. Materials and Methods

### 2.1. Materials

3-Bromo-1-propyne (BrP), sodium azide (NaN_3_), copper(II) sulfate pentahydrate (CuSO_4_·5H_2_O), and sodium (+)-ascorbate (NaAsc) were purchased from FUJIFILM Wako Pure Chemical Co. (Osaka, Japan). Poly(ethylene glycol) (PEG) monomethyl ether (*M*_n_ ~750 and 2000, EG18 and EG45, respectively) and *N*,*N*-dimethylformamide (DMF) (≥99.9%) were purchased from Sigma-Aldrich (St. Louis, MO, USA). Solvents (e.g., acetone, toluene, dichloromethane, diethyl ether, and DMF), were purchased from FUJIFILM Wako Pure Chemical Co. (Osaka, Japan). Water was purified with a Millipore Milli-Q system. All the reagents were used without further purification. 

### 2.2. Measurements

^1^H NMR spectra were recorded on a JEOL JNM ECA500 spectrometer using CDCl_3_ or dimethyl sulfoxide-*d*_6_ (DMSO-*d*_6_) as a solvent. Chemical shifts were referenced to the solvent value (7.26 and 2.50 ppm for CDCl_3_ and DMSO-*d*_6_, respectively). Size exclusion chromatography (SEC) measurements were carried out at 40 °C on a TOSOH HLC-8320GPC equipped with a TOSOH TSKgel SuperAWM-H column, using dimethyl sulfoxide (DMSO) containing 10 mM LiBr as eluent at a flow rate of 0.4 mL min^−1^. Molecular weights were calibrated with PEG and poly(ethylene oxide) (PEO) standards (Scientific Polymer Products, Inc., Ontario, NY, USA). All sample solutions for SEC were filtrated with a DISMIC-13JP PTFE 0.50 μm filter (ADVANTEC, Tokyo, Japan) just prior to injection. UV–Vis absorption spectra were collected on a HITACHI U-4100 spectrophotometer by using a 1.0 cm path length quartz cuvette at room temperature. Fluorescence measurements were performed on a HITACHI F-2500 equipped with a single cuvette reader at room temperature. The slit widths for both the excitation and emission sides were kept at 5.0 nm during measurement. Absolute quantum yields (Φ) were evaluated with a Hamamatsu Photonics C9920-02 absolute photoluminescence (PL) quantum yield measurement system. Pulse field gradient spin-echo (PGSE) NMR data were obtained on a Bruker AVANCE 700 NMR spectrometer at 30 °C using *N*,*N*-dimethylformamide-*d*_7_ (DMF-*d*_7_) as a solvent. The bipolar pulse pair stimulated echo (BPPSTE) sequence was applied [31,32,33]. The strength of pulsed gradients (*g*) was increased from 0.96 to 47.2 gauss cm^−1^. The time separation of pulsed field gradients (Δ) and their duration (δ) were 0.05 and 0.004 s, respectively. The sample was not spun, and the airflow was disconnected. The shape of the gradient pulse was rectangular, and its strength was varied automatically during the course of the experiments.

### 2.3. Preparation of Monopropargyl-Terminated Poly(ethylene glycol) (EGm-P)

A typical procedure for EG*m*-P is described below [34].

EG45 (15.02 g, 7.5 mmol) was added in toluene (50 mL) with stirring at room temperature for 0.5 h to make sure that all the EG45 was dissolved in toluene. Then NaOH (1.83 g, 45.8 mmol) and BrP (1.2 mL, 15.0 mmol) were added. After stirring for 4 h, BrP (1.2 mL, 15.0 mmol) and NaOH (1.80 g, 45.0 mmol) were further added to the reaction mixture with continuous stirring for 26 h. The colorless precipitate was removed from the reaction mixture by filtration, and the solvent was then removed under reduced pressure. The residue was dissolved in an aqueous solution of NaCl (5.1 wt %, 100 mL). Then the product was extracted from the aqueous solution with CH_2_Cl_2_ (3 × 50 mL). The organic layers were combined. The combined organic phase was washed with an aqueous solution of NaCl (5.1 wt %, 2 × 100 mL). After drying the organic phase with anhydrous MgSO_4_, the MgSO_4_ was removed by filtration. The solvent was removed under reduced pressure to obtain crude product. Finally, the product was purified by reprecipitation with CH_2_Cl_2_ and hexane twice. The final product was recovered as pale yellow gel after drying under vacuum at 45 °C for 24 h; yield was 13.1 g (86.4%).

### 2.4. Preparation of EGm-b-APn Block Copolymers

A typical example of the preparation of diblock copolymers of PEG and dense 1,2,3-triazole blocks (EG*m*-*b*-AP*n*, where *m* and *n* denote the degrees of polymerization (DP)) is described below.

A solution of EG45-P (2.52 g, 1.25 mmol) in DMF (5 mL), CuSO_4_·5H_2_O (0.030 g, 0.125 mmol), and NaAsc (0.050 g, 0.25 mmol) were added to a 100 mL flask under a nitrogen atmosphere. The mixture was stirred for 5 min. Then, NaN_3_ (1.95 g, 30 mmol) and BrP (2.8 mL, 25 mmol, 80 wt % in toluene) were added to the reaction mixture. The reaction mixture was warmed with an oil bath at 80 °C with stirring. After 72 h, the reaction mixture was added to DMF (50 mL). The precipitate was removed by filtration. The solution was then passed through a neutral Al_2_O_3_ column. The volatile fraction was removed under reduced pressure. The polymer obtained, EG45-*b*-AP22, was purified by reprecipitation by using pairs of solvents (i.e., a DMF/CH_2_Cl_2_ (1/20, *v*/*v*) mixed solvent and acetone three times). The final product was dried at 80 °C under vacuum; brown solid, 1.2 g, 34.9%.

### 2.5. Density Functional Theory (DFT) Calculations

To investigate the highest-occupied and lowest-unoccupied molecular orbitals (HOMO and LUMO, respectively), DFT calculations were carried out for model systems of EG*m*-*b*-AP*n* using the Gaussian 09 program [35]. In all the calculations, DFT with B3LYP functional was used, and 6-31+G(d) basis sets were applied for the hydrogen, carbon, nitrogen, and oxygen atoms. All the geometries of the model systems were fully optimized.

## 3. Results and Discussion

### 3.1. Preparation of EGm-b-APn Block Copolymers

**Scheme 1:**
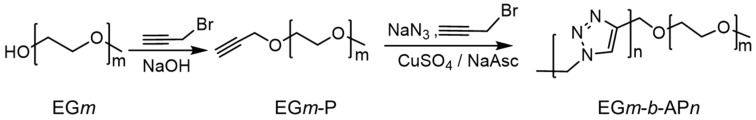
Synthetic route of EG*m*-P and EG*m*-*b*-AP*n*.

Samples of EG*m*-*b*-AP*n* were prepared by CuAAC polymerization of 3-azido-1-propyne (AP) in the presence of PEG modified with a propargyl group at one end (EG*m*-P) (Scheme 1). Since the reaction mixture of this procedure contained not only EG*m*-*b*-AP*n* diblock copolymer but also AP homopolymer and the remaining EG*m*-P, EG*m*-*b*-AP*n* samples were purified by reprecipitation using an appropriate combination of good and poor solvents to remove the homopolymers. Table 1 summarizes the basic characteristics of the four EG*m*-*b*-AP*n* samples obtained. These samples were characterized by SEC and ^1^H NMR as can be seen in Figures S1–S3 in the Supporting Information. Our previous work has demonstrated that AP homopolymer is insoluble in all the common solvents examined, including DMF, DMSO, *N*-methylpyrrolidone (NMP), methanol, acetone, THF, chloroform, and toluene [21,22]. Thus, the solubility of the EG*m*-*b*-AP*n* diblock copolymer samples was examined, and the results are summarized in Table 2. Due to the PEG block, the EG*m*-*b*-AP*n* samples were soluble in many common solvents. The samples were especially well soluble in polar organic solvents. 

### 3.2. Emission Properties of EGm-b-APn Block Copolymers

**Figure 1:**
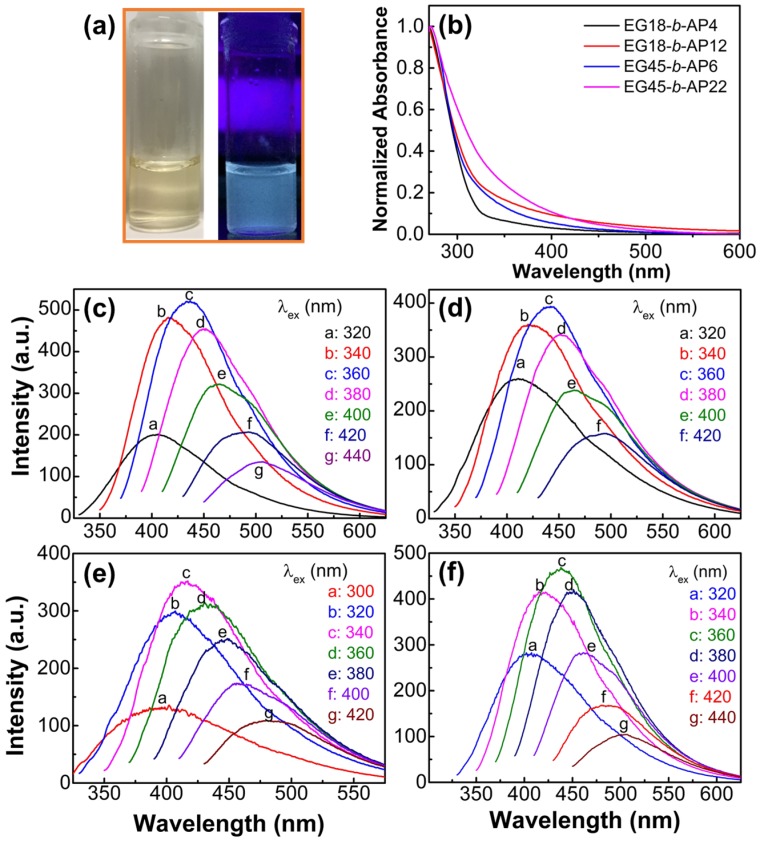
Photographs of EG45-*b*-AP22 in DMF at 1.0 g L^−1^ under visible (**left**) and UV (*λ* = 365 nm) light (**right**) (**a**), UV–Vis absorption spectra normalized at 270 nm for 0.30 g L^−1^ solutions of EG*m*-*b*-AP*n* in DMF (**b**), and steady-state fluorescence spectra for 1.0 g L^−1^ solutions of EG18-*b*-AP4 (**c**), EG18-*b*-AP12 (**d**), EG45-*b*-AP6 (**e**), and EG45-*b*-AP22 (**f**) in DMF with excitation at varying wavelengths.

When a solution of EG*m*-*b*-AP*n* in DMF was measured by dynamic light scattering in order to characterize the polymer chains in the molecularly dispersed state, emission from the solution was observed. Thus, the photophysical behavior of solutions of the EG*m*-*b*-AP*n* samples in DMF was examined by absorption and steady-state fluorescence spectroscopy. As can be seen in Figure 1a, a solution of EG45-*b*-AP22 in DMF emits blue fluorescence under irradiation with 360 nm UV light. Figure 1b displays UV–Vis absorption spectra for solutions of the EG*m*-*b*-AP*n* samples in DMF. These spectra exhibit absorption in the wavelength range of 270–450 nm, which is ascribable to the dense 1,2,3-triazole block. It should be noted here that EG45-*b*-AP22 shows absorption bands in the near UV and visible regime of 300–500 nm, which are stronger compared to that for EG45-*b*-AP6. This observation indicates that a longer dense 1,2,3-triazole block absorbs light of a longer wavelength. Figure 1c–f indicate steady-state fluorescence spectra for DMF solutions of EG*m*-*b*-AP*n* with excitation at varying wavelengths from 300 to 440 nm. These spectra exhibit that the solutions emit fluorescence of a wavelength increasing from UV to green with increasing excitation wavelength. As can be seen in Figures S4 and S5 in Supporting Information, solutions of EG*m*-*b*-AP*n* in acetonitrile and water also emit fluorescence depending on the excitation wavelength. This may be because the EG*m*-*b*-AP*n* samples possess a rather broad molecular weight distribution, and AP*n* blocks of different lengths are excited at different wavelengths [36,37]. The excitation and emission bands of AP*n* block are dependent on DP, i.e., *n*. Since EG*m*-P did not emit fluorescence [38,39], it is likely that the emission observed is from the dense 1,2,3-triazole block. The absolute fluorescence quantum yields were evaluated for the EG*m*-*b*-AP*n* samples in DMF, acetonitrile, and water. As listed in Table 3, the quantum yield ranges from 1.4–3.4%, indicating that the quantum yields in DMF are higher than those in the other solvents.

**Figure 2:**
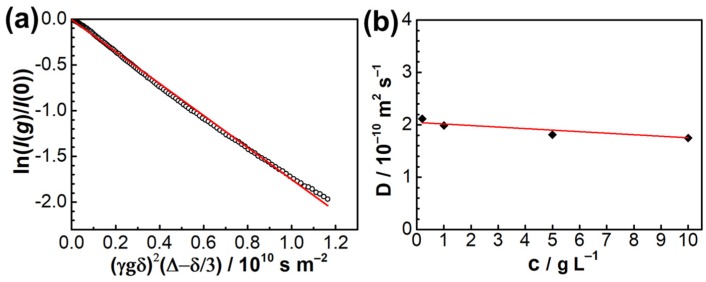
Pulse field gradient spin-echo (PGSE) NMR data for 10.0 g L^−1^ solution of EG45-*b*-AP22 in DMF-*d*_7_ (**a**) and the concentration dependencies of *D* (**b**).

Recently, aggregation-induced emission (AIE) has attracted increasing interest from researchers because of its potential application for molecular sensors [40]. Some examples of AIE from polymers possessing no aromatic moieties have been reported. It is important to know whether or not the emission observed from EG*m*-*b*-AP*n* is AIE. The association behavior of EG*m*-*b*-AP*n* was preliminarily investigated in DMF by PGSE NMR [41]. The PGSE NMR data are presented in Figure 2a. The intensity data practically obey a straight line at four different concentrations, indicative of a unimodal distribution. The unimodal distribution is confirmed by diffusion-ordered spectroscopy (DOSY) data as can be seen in Figure S4 in the Supporting Information. From the slopes of straight lines, apparent diffusion coefficients (*D*) were evaluated and plotted in Figure 2b against the polymer concentration. The diffusion coefficient (*D*_0_) was determined by extrapolation to zero concentration. Using the Einstein–Stokes equation, the hydrodynamic radius (*R*_H_) was calculated to be 1.35 nm for EG45-*b*-AP22. The *R*_H_ value is almost the same as that for PEG of the same molecular weight, indicating that most of EG45-*b*-AP22 chains are molecularly dispersed in DMF. As discussed in a later subsection, the fluorescence intensity decreased markedly when a small amount of water was added to a solution of EG*m*-*b*-AP*n* in DMF to induce aggregation because water is a rather poor solvent for EG*m*-*b*-AP*n*. This observation confirms that the emission observed for DMF solutions is from EG*m*-*b*-AP*n* in the molecularly dispersed state.

**Figure 3:**
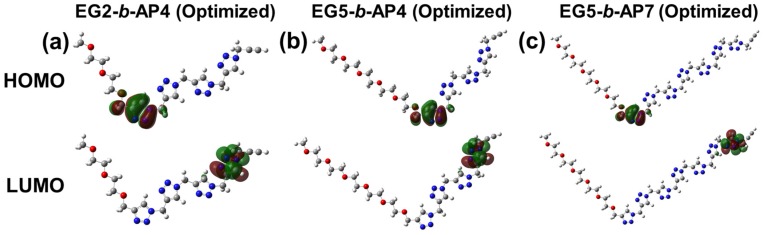
Optimized structures as well as the highest-occupied molecular orbitals (HOMO) and lowest-unoccupied molecular orbitals (LUMO) for EG2-*b*-AP4 (**a**), EG5-*b*-AP4 (**b**), and EG5-*b*-AP7 (**c**) obtained by DFT calculations using a B3LYP/6-31+G(d) basis set with a Gaussian’09 program (blue: N; red: O; grey: C; white: H).

To understand the detail mechanism of the fluorescence observed, the HOMO and LUMO of EG*m*-*b*-AP*n* were estimated by preliminary DFT calculations. As shown in Figure 3, the HOMO is located on the 1,2,3-triazole unit directly connecting the PEG block. On the other hand, the LUMO is located on the terminal 1,2,3-triazole unit. These observations indicate that the emission is from the dense 1,2,3-triazole block. The absorption and fluorescence wavelengths were also evaluated by DFT calculation, as listed in Table 4. The length of PEG block has no or only a little effect on the absorption and fluorescence wavelengths. On the other hand, as the degree of polymerization of AP*n* block is increased from 4 to 7, the absorption and fluorescence wavelengths become longer (i.e., a red shift). The red shift is larger for fluorescence than that for absorption. These data indicate that the absorption and fluorescence wavelengths depend on the length of AP*n* block. It is noteworthy that the fluorescence wavelength calculated agrees well with the fluorescence maximum observed [42,43]. On the basis of these observations, it is concluded that the emission from solutions of EG*m*-*b*-AP*n* is not AIE, and the fluorescence is from the dense 1,2,3-triazole blocks in the molecularly dispersed state.

**Figure 4:**
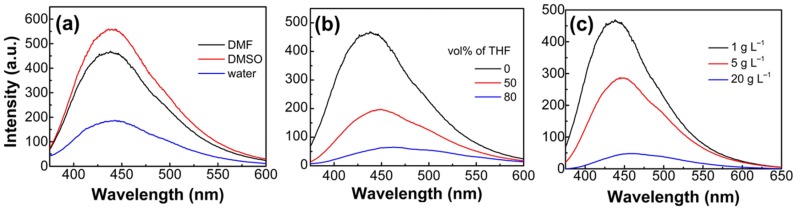
Steady-state fluorescence spectra for solutions of EG45-*b*-AP22 (1.0 g L^−1^) in water, DMF, and DMSO (**a**), in mixed solvent of DMF and THF with different THF contents (**b**), and in DMF at different concentrations with excitation at 360 nm (**c**).

The effects of the solvent and polymer concentration on fluorescence behavior were examined. Figure 4a displays fluorescence spectra for EG45-*b*-AP22 in different solvents with excitation at 360 nm. The block copolymer shows an emission band depending on the solvent used. When a small amount of water was added to the solution of EG45-*b*-AP22 in DMF, the fluorescence intensity decreased remarkably. As can be seen in Table 2, water is not a good solvent for EG45-*b*-AP22 compared to DMF and DMSO. Since our preliminary light scattering data indicated that EG45-*b*-AP22 forms aggregates in water (not shown), it is likely that the aggregation of EG45-*b*-AP22 quenches fluorescence. Figure 4b exhibits fluorescence spectra for EG45-*b*-AP22 in mixed solvent of DMF and THF, a poor solvent, indicating that the fluorescence intensity decreases by increasing the THF content. As can be seen in Figure 4b, the fluorescence intensity decreases by increasing the polymer concentration from 1.0 to 20 g L^−1^. These observations confirm that aggregation quenches fluorescence.

### 3.3. Metal Ion-Responsive Emission of EGm-b-APn Block Copolymers

**Figure 5:**
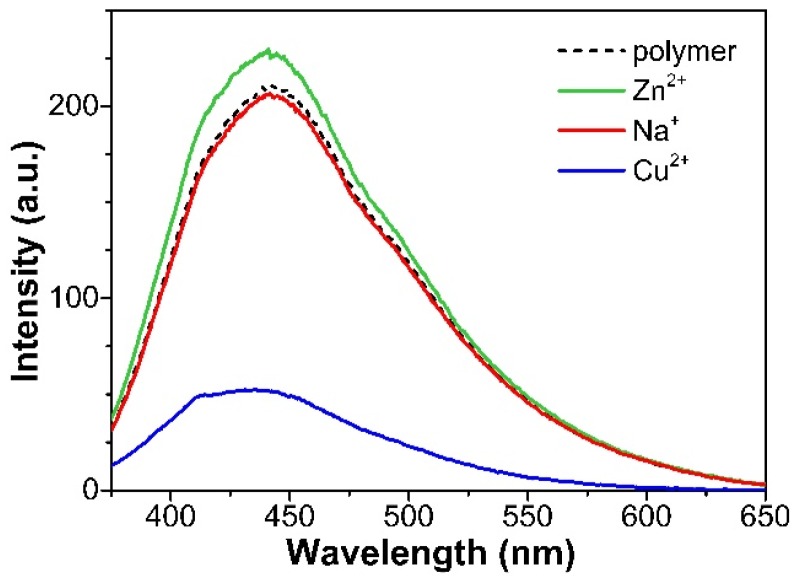
Steady-state fluorescence spectra for a 1.0 g L^−1^ aqueous solution of EG18-*b*-AP10 with excitation at 360 nm in the absence and the presence of 0.10 M Na^+^, 0.10 M Zn^2+^, or 0.005 M Cu^2+^.

As described in the introduction part, it is known that 1,2,3-triazole moieties can act as metal ligands [16,44]. It is thus expected that the fluorescence behavior of EG*m*-*b*-AP*n* diblock copolymers should be responsive to the metal ion added. In this study, the effect of the addition of Na^+^, Zn^2+^, or Cu^2+^ was investigated. Figure 5 shows fluorescence spectra for an aqueous solution of EG18-*b*-AP10 in the absence and presence of Na^+^, Zn^2+^, or Cu^2+^. In the presence of Na^+^ or Zn^2+^, the fluorescence spectra are almost the same as that in the absence. In the presence of Cu^2+^, however, the fluorescence intensity is markedly weaker. This may be because dense 1,2,3-triazole moieties coordinate Cu^2+^ ions and fluorescence is quenched through the heavy metal effect. These observations indicate that the fluorescence of EG*m*-*b*-AP*n* can be applicable to a molecular sensor of metal ions.

## 4. Conclusions

In conclusion, we have synthesized dense triazole-based block copolymers, EG*m*-*b*-AP*n*, and studied their solubility and photophysical characterization in solution state. The incorporation of EG*m* block significantly enhanced the solubility of bock copolymers in common polar solvents. Their solubility decreases by increasing the length of AP*n* block. Significantly, without attachment of any extra fluorophore, the as-prepared block copolymers emit fluorescence in solution state. The emission of block copolymers is tunable from ultraviolet to green fluorescence by changing the excitation wavelength presumably because a longer AP*n* block is excited at a longer wavelength. PGSE NMR analysis and theoretical calculation indicate that the copolymers are molecularly dispersed in DMF-*d*_7_ and AP*n* block is an intrinsic fluorophore. It is noteworthy that the emission of block copolymer is responsive to metal ions because of the interaction with dense 1,2,3-triazole blocks dependent on the ionic species. This work will afford a new aspect for fabricating novel emissive triazole-based functional materials.

## Figures and Tables

**Table 1:** Molecular characteristics of EG*m*-*b*-AP*n* samples prepared by copper(I)-catalyzed azide–alkyne cycloaddition (CuAAC) polymerization.

Polymer Code ^a^	*M*_n_ × 10^−3 b^	*M*_n_ × 10^−3 c^	*M*_w_/*M*_n_^c^
EG18-*b*-AP4	1.1	1.5	1.26
EG18-*b*-AP12	1.8	3.3	1.98
EG45-*b*-AP6	2.5	2.7	1.40
EG45-*b*-AP22	3.6	2.5	2.37

^a^ The numbers denote DP determined by ^1^H NMR spectroscopy. ^b^ Determined by ^1^H NMR. ^c^ Determined by size exclusion chromatography (SEC) in DMSO at 40 °C calibrated with poly(ethylene glycol) (PEG) and poly(ethylene oxide) (PEO) standards.

**Table 2:** Results of solubility test for EG*m*-*b*-AP*n* samples.^1^

Solvent	EG18-*b*-AP4	EG18-*b*-AP12	EG45-*b*-AP6	EG45-*b*-AP22
water	++	+	++	+
methanol	+	–	++	+
acetic acid	++	++	++	++
NMP	++	++	++	++
DMSO	++	++	++	++
DMF	++	++	++	++
acetonitrile	++	+	++	+
acetone	++	–	+	–
dicholromethane	++	++	++	+
chloroform	++	++	++	+
pyridine	++	++	++	+
THF	+	–	+	–
ethyl acetate	+	–	–	–
toluene	–	–	–	–

^1^ “++”, “+”, and “−” denote well soluble (≥1 wt %), slightly soluble (<1 wt %), and insoluble, respectively.

**Table 3:** Fluorescence quantum yields (Φ) for the EG*m*-*b*-AP*n* samples in DMF, acetonitrile, and water (1.0 g L^−1^).

Polymer Code	Φ		
	In DMF	In Acetonitrile	In Water
EG18-*b*-AP4	0.034	0.020	0.018
EG18-*b*-AP12	0.020	—	—
EG45-*b*-AP6	0.029	0.019	0.014
EG45-*b*-AP22	0.024	—	—

**Table 4:** Time-dependent DFT (TDDFT)-predicted properties of models of EG*m*-*b*-AP*n*.

Model	Abs. ^1^/nm	Emission ^2^/nm
EG2-*b*-AP4	231.1	456.6
EG5-*b*-AP4	231.4	455.7
EG5-*b*-AP7	237.1	473.9

^1^ Energy (TDDFT) using the optimized structure in ground state. ^2^ Optimization (TDDFT, root = 1) using the optimized structure in ground state.

## Supplementary Materials

The following are available online at https://www.mdpi.com/2073-4360/11/7/1086/s1, Figure S1: SEC analysis of EG*m*-P and EG*m*-*b*-AP*n* samples, Figure S2: ^1^H NMR spectra of EG18-*b*-AP*n* in DMSO-*d*_6_ (500 MHz, 25 °C), Figure S3: ^1^H NMR spectra of EG45-*b*-AP*n* in DMSO-*d*_6_ (500 MHz, 25 °C), Figure S4: Steady-state fluorescence spectra for 1.0 g L^−1^ solutions of EG18-*b*-AP4 in acetonitrile (**a**) and water (**b**), Figure S5: Steady-state fluorescence spectra for 1.0 g L^−1^ solutions of EG45-*b*-AP6 in acetonitrile (**a**) and water (**b**), Figure S6: DOSY spectrum for a 10.0 g L^−1^ solution of EG45-*b*-AP22 in DMF-*d*_7_ at 298 K.
