# Supplementary material for: Emission Properties of Diblock Copolymers Composed of Poly(ethylene glycol) and Dense 1,2,3-Triazole Blocks
Source: Polymers (Basel). 2019 Jun 26;11(7):1086. doi: 10.3390/polym11071086 (PMC6681435; doi:10.3390/polym11071086)
Supplement: Supplementary file 1 [file polymers-11-01086-s001.zip › polymers-531550-SI.pdf]

Supporting Information for

Emission Properties of Diblock Copolymers Composed of Poly(ethylene glycol) and Dense 1,2,3-Triazole Blocks

Yanqiong Yang [1], Asami Mori [1] and Akihito Hashidzume [1,*]

[1] Department of Macromolecular Science, Graduate School of Science, Osaka University; hashidzume@chem.sci.osaka-u.ac.jp

* Correspondence: hashidzume@chem.sci.osaka-u.ac.jp; Tel.: +81-6-6850-8174

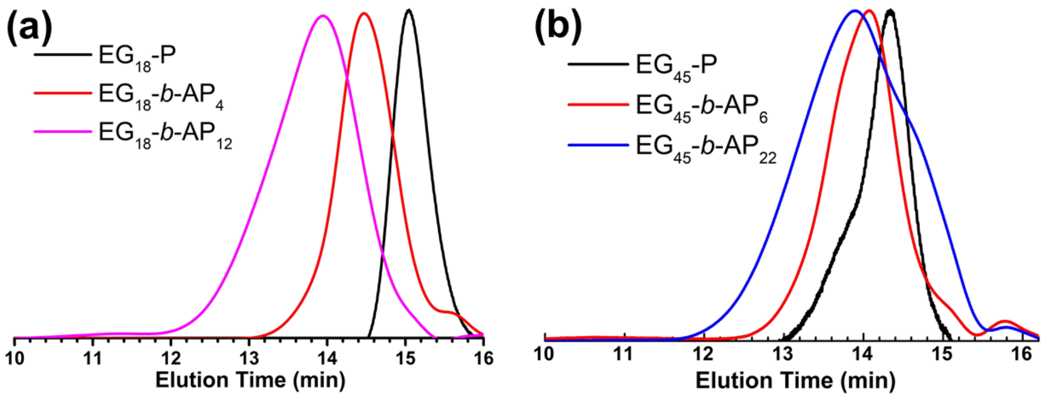

Figure S1. SEC analysis of EG*m*-P and EG*m*-*b*-AP*n* samples.

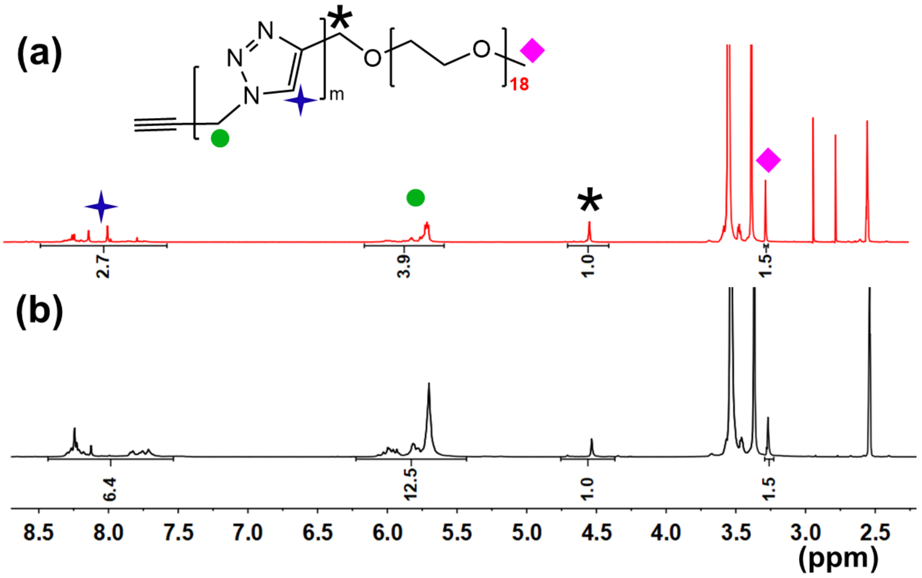

Figure S2. ^{1}H NMR spectra of EG18-*b*-AP*n* in DMSO-d_6 (500 MHz, 25 °C).

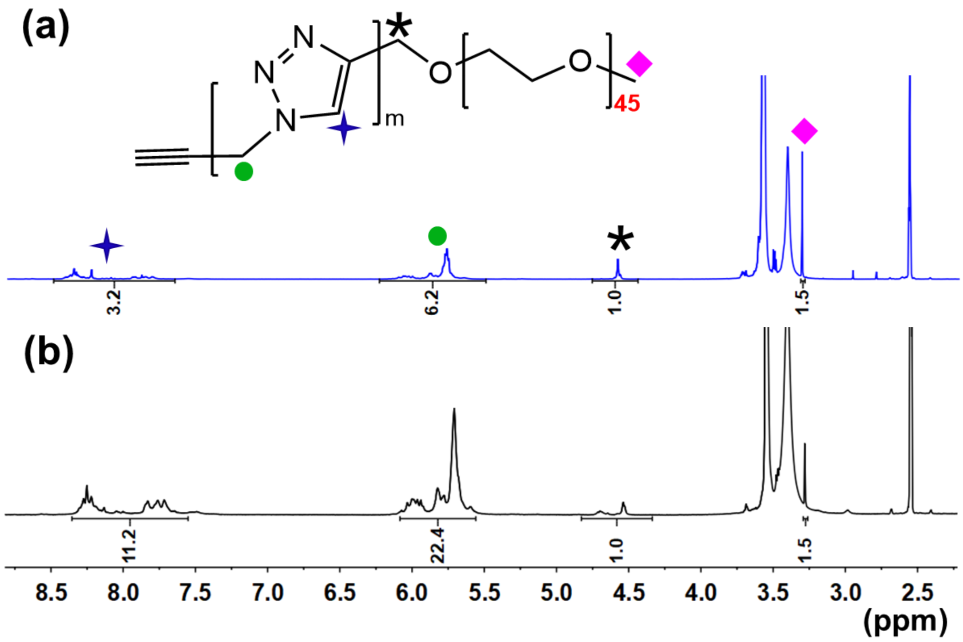

Figure S3. ^{1}H NMR spectra of EG45-*b*-AP*n* in DMSO-d_6 (500 MHz, 25 °C).

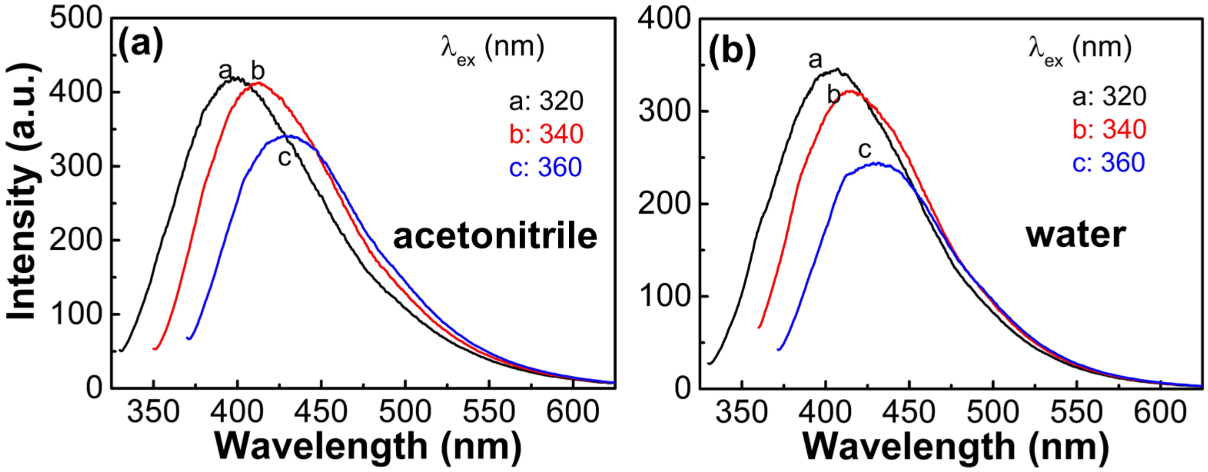

Figure S4. Steady-state fluorescence spectra for 1.0 g L^{-1} solutions of EG18-*b*-AP4 in acetonitrile (a) and water (b).

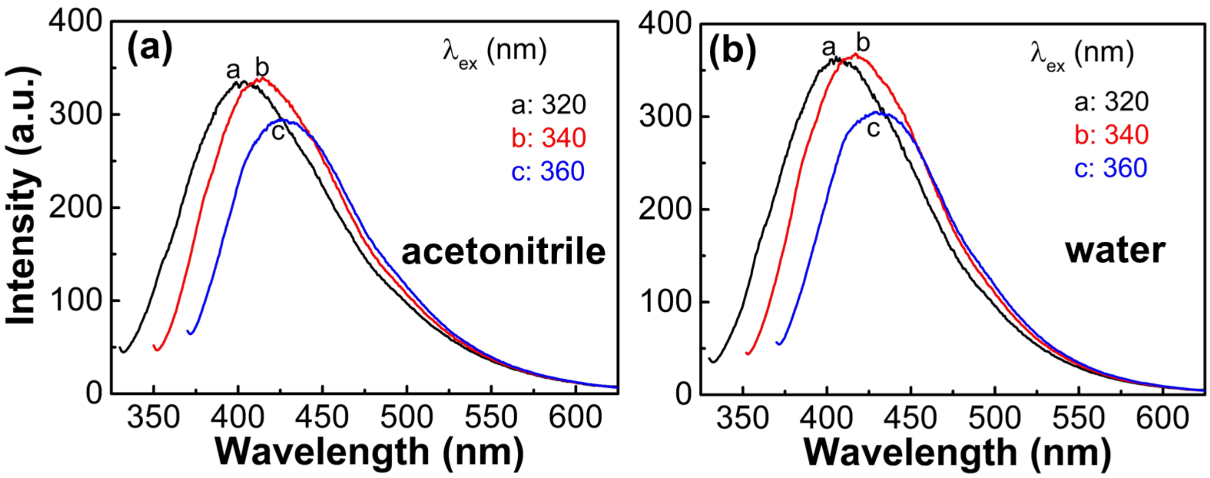

Figure S5. Steady-state fluorescence spectra for 1.0 g L^{-1} solutions of EG45-*b*-AP6 in acetonitrile (a) and water (b).

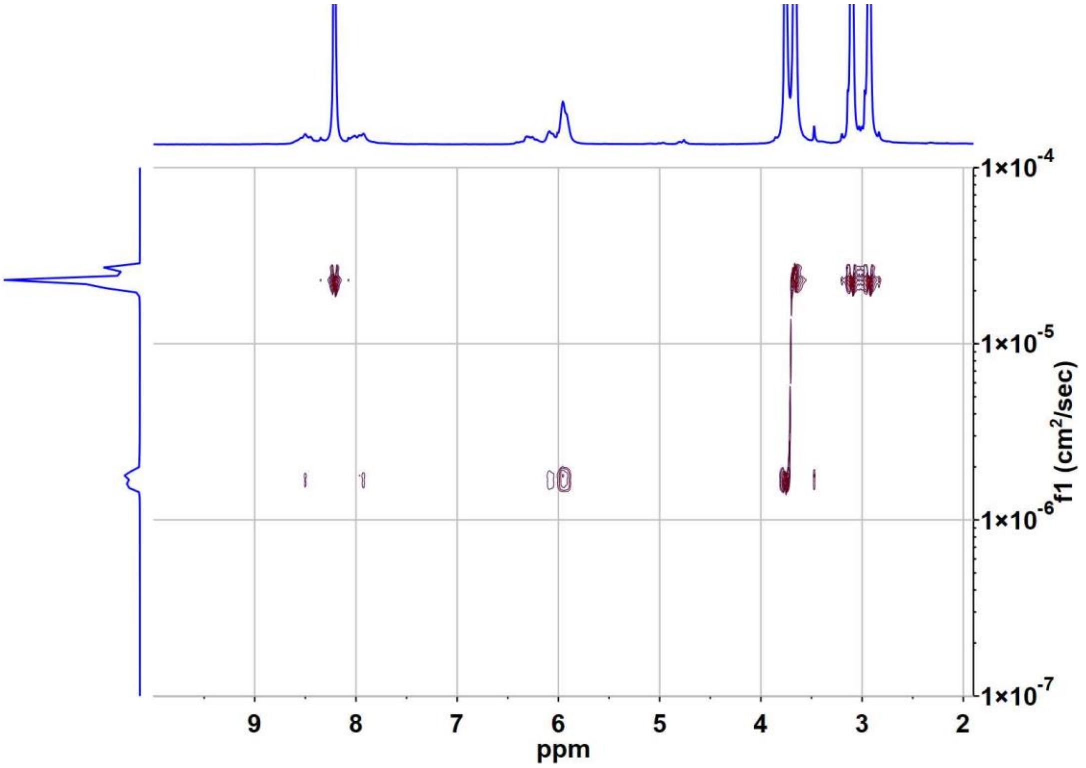

Figure S6. DOSY spectrum for a 10.0 g L^{-1} solution of EG45-*b*-AP22 in DMF-d_7 at 298 K.
